# Small but Mighty: A Microfluidic Biofuel Cell-Based Biosensor for the Determination of Ethanol

**DOI:** 10.3390/molecules30030673

**Published:** 2025-02-03

**Authors:** Jirawan Monkratok, Pattanaphong Janphuang, Kantapat Chansaenpak, Sireerat Lisnund, Vincent Blay, Piyanut Pinyou

**Affiliations:** 1 School of Chemistry, Institute of Science, Suranaree University of Technology, 111 University Ave., Nakhon Ratchasima 30000, Thailand; jirawan@g.sut.ac.th; 2 Synchrotron Light Research Institute (Public Organization), 111 University Ave., Nakhon Ratchasima 30000, Thailand; pattanaphong@slri.or.th; 3 National Nanotechnology Center, National Science and Technology Development Agency, Thailand Science Park, Pathum Thani 12120, Thailand; kantapat.cha@nanotec.or.th; 4 Department of Applied Chemistry, Faculty of Science and Liberal Arts, Rajamangala University of Technology Isan, 744, Suranarai Rd., Nakhon Ratchasima 30000, Thailand; sireerat.in@rmuti.ac.th; 5 Department of Microbiology and Environmental Toxicology, University of California at Santa Cruz, Santa Cruz, CA 95064, USA

**Keywords:** microfluidics, biofuel cell, ethanol, alcohol dehydrogenase, horseradish peroxidase, biosensor

## Abstract

We developed a membraneless-microfluidic biofuel cell (MBFC) for the quantification of ethanol. The system employs anolyte and catholyte solutions, each containing a biocatalyst and redox mediator. The laminar flow conditions in the microfluidic chip minimize the mixing between anolyte and catholyte and obviate the need for a membrane to separate them. When ethanol is added to the anolyte, alcohol dehydrogenase (ADH) catalyzes its oxidation to acetaldehyde, releasing electrons to the anode. On the cathode, electrons are transferred to horseradish peroxidase (HRP), which reduces hydrogen peroxide in the catholyte to water. We optimized key design factors and operating conditions. We also studied the incorporation of glycerol as a viscosity modifier, which improved the power and current density supplied by the MBFC, with a maximum power output of 307 µW cm^−2^ and an open circuit voltage of 0.733 V. The proposed ethanol/hydrogen peroxide MBFC was successfully applied as a biofuel cell-based sensor for the quantification of ethanol in a commercial liquor.

## 1. Introduction

Biofuel cells (BFCs) can enable sensing and power supply solutions and are particularly well suited to support the Sustainable Development Goals (SDG) [1], including sustainable industrialization (SDG 9), healthcare (SDG 3), and clean energy (SDG 7) [2,3]. BFCs utilize redox enzymes as biocatalysts to oxidize fuels, such as glucose or ethanol, at the anode [4,5]. This process generates electrons, which are transferred through an external circuit to the cathode as an electric current. The cathode can employ other enzymes to reduce an oxidant, such as oxygen or hydrogen peroxide, closing the circuit [6]. BFCs offer several advantages, including the use of biodegradable materials, operation under mild conditions, and potential integration with biological systems [7,8,9,10].

A microfluidic biofuel cell (MBFC) is a biofuel cell that utilizes flows within microchannels to produce electrical energy [11]. MBFCs can be classified into membrane-MBFCs and membraneless-MBFCs, depending on whether the fuel (anolyte) and oxidant (catholyte) are separated or not by a membrane [12]. The membrane prevents mixing but complicates fabrication and introduces an additional ohmic drop. Membraneless-MBFCs rely on laminar flows to minimize mixing of the anolyte and catholyte, which may contain enzymes in solution. The performance of a membraneless-MBFC is affected by its design [13], by diffusional cross-over at the liquid–liquid interface [14], and by diffusion and reaction rates on the electrodes [15], among other factors. The applications of membraneless-MBFC are expanding, including powering microelectronics [16] and sensing [17,18]. Biofuel cell-based self-powered biosensors utilize the electricity produced by a BFC and avoid the need for an external power supply [19,20], facilitating miniaturized and implantable devices [21].

Ethanol is an appealing fuel for BFCs given its low toxicity, high energy density, and potential production from biomass [22,23]. Honorio Franco et al. have reviewed recent progress in ethanol BFCs [24] and some designs are compared in Table 2. The use of enzymes enables cleaner oxidation than Pt-based catalysts, which suffer from electrode fouling [25]. Ethanol BFCs employ alcohol dehydrogenase (ADH) or alcohol oxidase (AOx) to catalyze ethanol oxidation [26,27]. ADH is most often used and requires NAD^+^ as a cofactor. NAD^+^ is unaffected by oxygen and offers lower anode potentials, which may enhance overall cell voltage [28,29,30]. The regeneration of NADH requires additional catalysts, such as toluidine blue O (TBO) [28] or methylene green (MG) [29,30]. Besides power generation, ethanol sensing has attracted great interest in diverse applications and samples, including sweat [27] and alcoholic beverages [31]. There has been considerable effort to increase the power output of ethanol BFCs [30,32] and MBFCs [33]. However, the potential of MBFCs for ethanol biosensing has not been previously investigated.

**Figure 1:**
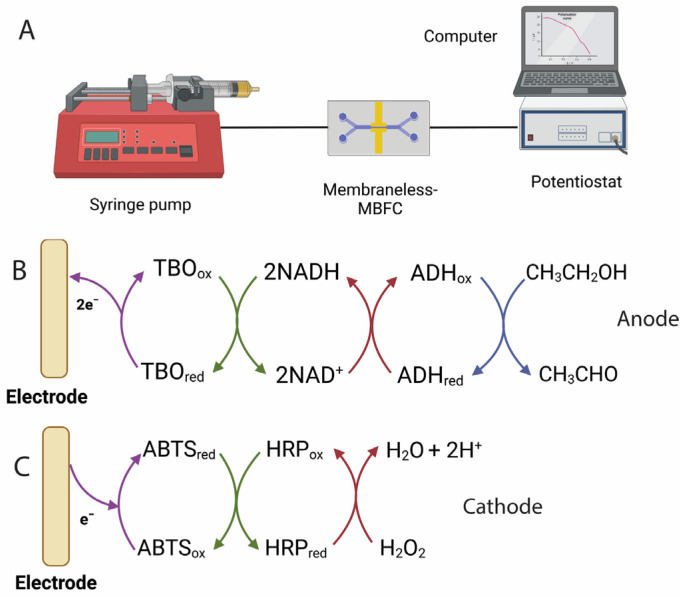
(**A**) A membraneless-MBFC system was built based on the oxidation of ethanol on the anode and the reduction of H_2_O_2_ on the cathode. (**B**) Mediated electron transfer (MET) at the anode, involving NAD-ADH as the enzyme, TBO as the mediator, and ethanol as the fuel. (**C**) MET at the cathode, involving HRP as the enzyme, ABTS as the mediator, and H_2_O_2_ as the oxidant.

In this work, we report an ethanol/hydrogen peroxide membraneless-MBFC biosensor based on a Y-shaped microfluidic device (Figure 1A). The microfluidic device is pressure driven using a syringe pump and operates under laminar flow conditions. At the anode, ethanol is oxidized by ADH in the presence of the redox mediator TBO. At the cathode, hydrogen peroxide is reduced by horseradish peroxidase (HRP) in the presence of the redox mediator 2,2′-Azino-bis-(3-ethylbenzothiazoline-6-sulfonic acid) (ABTS). We chose HRP due to its high redox potential, which might boost cell voltage and power output. We dissolved the mediators and enzymes directly in electrolyte to ensure a simple and robust operation. The electrochemical performance of the device, including its power output and cell voltage, was studied under different conditions. The effect of electrolyte viscosity was also investigated. The MBFC delivers high open-circuit voltage, requires a small volume of sample, and can be used as a biofuel cell-based ethanol biosensor with a digital multimeter as a readout, eliminating the need for a potentiostat for ethanol analysis.

## 2. Results and Discussion

### 2.1. Electrochemical Study of Enzymes and Redox Mediators

The electron transfer pathways for the BFC are illustrated in Figure 1. In the anode section (Figure 1B), the ADH enzyme catalyzes the conversion of ethanol to acetaldehyde, while the redox cofactor NAD^+^ is reduced to NADH. With NAD-dependent enzymes, the use of a redox mediator is necessary to regenerate NAD^+^ and facilitate electron transfer. TBO has been widely used as a redox mediator for the regeneration of NADH in electrodes modified with NAD-dependent enzymes [34] and it was also employed in this study. The reduced TBO transfers electrons to the electrode, after which the oxidized TBO is regenerated and ready for reuse. On the cathode side (Figure 1C), the biochemical process involves the reduction of H_2_O_2_, catalyzed by HRP, with ABTS acting as the redox mediator.

**Figure 2:**
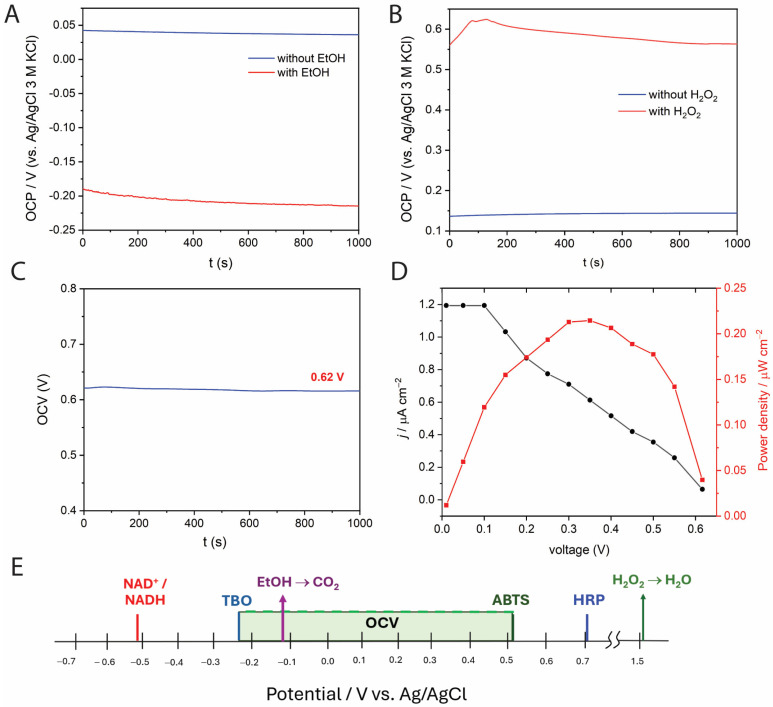
Open-circuit potential (OCP) measurement of phosphate buffer pH 7.4 containing (**A**) 0.24 mg/mL ADH, 1.1 mM TBO, 2.5 mM NAD^+^ and 0.57 mM EtOH, and (**B**) 0.5 mg/mL HRP, 5.0 mM ABTS, and 10 mM H_2_O_2_. OCP measurements were conducted with a 3-electrode setup. (**C**) Open-circuit voltage (OCV) of the BFC assembled in a 2-compartment cell separated by a Nafion™ (Kuala Lumpur, Malaysia) 117 membrane in the presence of 0.57 mM EtOH and 10 mM H_2_O_2_ and air-equilibrated. (**D**) Voltage–power density and voltage–current density profiles of the BFC in a 2-compartment electrochemical cell. Each compartment was 10.0 mL and operated under air-equilibrated conditions. (**E**) Formal potentials of the redox species associated with the oxidation of ethanol by NAD-ADH (anode) and the reduction of H_2_O_2_ by HRP (cathode) compared with the thermodynamic potentials of the anode and cathode and the possible operating open-circuit potential.

Before investigating the microfluidic setup, we studied the electrochemical behavior of the individual anode and cathode. The results are shown in Figure 2. For the anode (Figure 2A), the OCP shifted from approximately 0.040 V without EtOH to −0.215 V with 0.57 M EtOH.

Although the redox potential of NAD^+^/NADH is around −0.50 V vs. Ag/AgCl [35], the use of TBO (formal potential = −0.21 V [36]) as an electron mediator is expected to introduce a potential loss. For the cathode (Figure 2B), the redox potential of HRP is about 0.7 V vs. Ag/AgCl at pH 7 [37]. Experimentally, the OCP increased from +0.140 V in the absence of H_2_O_2_ to +0.563 V when present at 10 mM. When the substrates were present in the electrolyte, both anode and cathode underwent potential shifts due to the catalytic reactions. The potential of the individual electrode is governed by the ratio of the activities of the redox species involved according to the Nernst equation [38]:

(1) $$E=E^{0}-\frac{RT}{nF}\ln\frac{\left(a_{red}\right)}{\left(a_{ox}\right)}$$

where E is the electrode potential, E^0^ is the formal potential of the electrode, n is the number of electrons involved in the reaction, and a is the chemical activity of the reduced and oxidized species.

Knowing the individual OCP for each electrode allows us to estimate the OCV of the BFC. The estimated OCV will be lower than the theoretical cell voltage (E^0^) due to losses (Equation (2)), including (i) activation loss (η_act_), (ii) ohmic loss (η_ohmic_), and (iii) concentration loss (η_conc_) [39].

(2) $$OCV=E^{0}-\eta_{act}-\eta_{ohmic}-\eta_{conc}$$

We performed the OCV measurements (Figure 2C) of the anode and the cathode in a compartmentalized cell separated by a Nafion™ 117 membrane using the same electrolyte concentration as in Figure 2A,B. The open-circuit voltage obtained was 0.62 V, which was lower than the theoretical value of ca. 0.78 V when considering the thermodynamic potentials (E^0^) of the redox mediators used in the anode and cathode. The use of a large volume cell (10 mL for each compartment) as well as large separation between electrodes (7.0 cm) could increase the ohmic loss. Polarization and power output curves were obtained (Figure 2D) and the maximum power output was 0.21 µW cm^−2^. Since large cell dimensions can decrease the operating cell voltage and its performance, the anolyte and catholyte were further studied and optimized in a microfluidic system.

**Figure 3:**
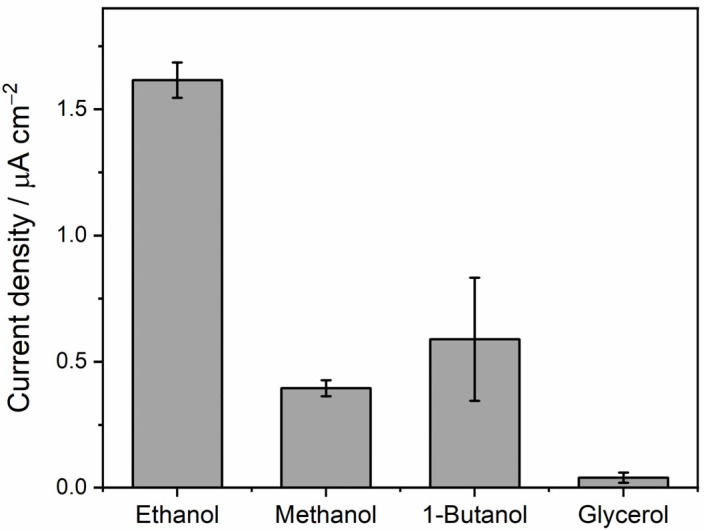
Current densities measured in the presence of different alcohols. Current density values were obtained from 1.0 M alcohols in phosphate buffer pH 7.4 containing 0.24 mg/mL ADH, 1.1 mM TBO, and 2.5 mM NAD^+^. Amperometry was conducted at a constant applied potential of 0.20 V vs. Ag using a 4 mm gold screen-printed electrode as the working electrode (n = 3 measurements).

To assess the suitability of alcohol dehydrogenase (ADH) as a biocatalyst in the biofuel cell-based ethanol biosensor, we investigated its substrate specificity using amperometry. We measured the enzyme activity on several primary alcohols, including ethanol, methanol, and n-butanol. As shown in Figure 3, ethanol yielded the highest current density, confirming it is the preferred substrate for ADH from *Saccharomyces cerevisiae*. This aligns with prior literature [40] showing that, while ADH can catalyze the oxidation of other primary alcohols like methanol and n-butanol, it exhibits a clear preference for ethanol. Importantly, the relatively low activity observed with other alcohols indicates that they would not significantly interfere with the function of an ethanol biosensor employing ADH.

We also investigated the influence of pH on ADH activity. Amperometric measurements of ethanol oxidation were conducted across a pH range of 5.8 to 9.0, revealing that ADH exhibited the highest current density at pH 8.0 (Figure S1). However, we decided to use a slightly lower pH of 7.4, for experimental convenience (commercial buffer is available at pH 7.4). Additionally, it has been reported that ADH stability is lower at pH 8 than at pH 7, and the buffering capacity of phosphate buffer is higherat pH 7.4 than at pH 8 (pK_2_ = 7.2) [41].

### 2.2. Assembly and Optimization of the Membraneless-MBFC

Several parameters can affect the performance of the membraneless-MBFC. In particular, the cross-mixing between the streams of anolyte and catholyte can limit the current density and OCV. Cross-mixing of anolyte and catholyte is reduced under laminar flow conditions, which are characterized by a low Reynolds number (Re < 10^3^) [42,43]. The Reynolds number (Equation (3)) is defined as:

(3) $$Re=\frac{\rho U_{av}D_{h}}{v}$$

where ρ is the fluid density (kg/m^3^), U_av_ is the average velocity (m/s), Dh is the hydraulic diameter (for a rectangular section D_h_ = $\frac{2ab}{a+b}$, where a is width and b is height, i.e., 100 and 1000 µm in our chip), and υ is the dynamic viscosity. Assuming a viscosity of 8.927 × 10^−4^ Pa·s (water at 25 °C), *Re* values are estimated to be 13.6, 33.9, and 67.6 for total flow rates 400, 1000, and 2000 µL/min, respectively, confirming the laminar flow regime.

**Figure 4:**
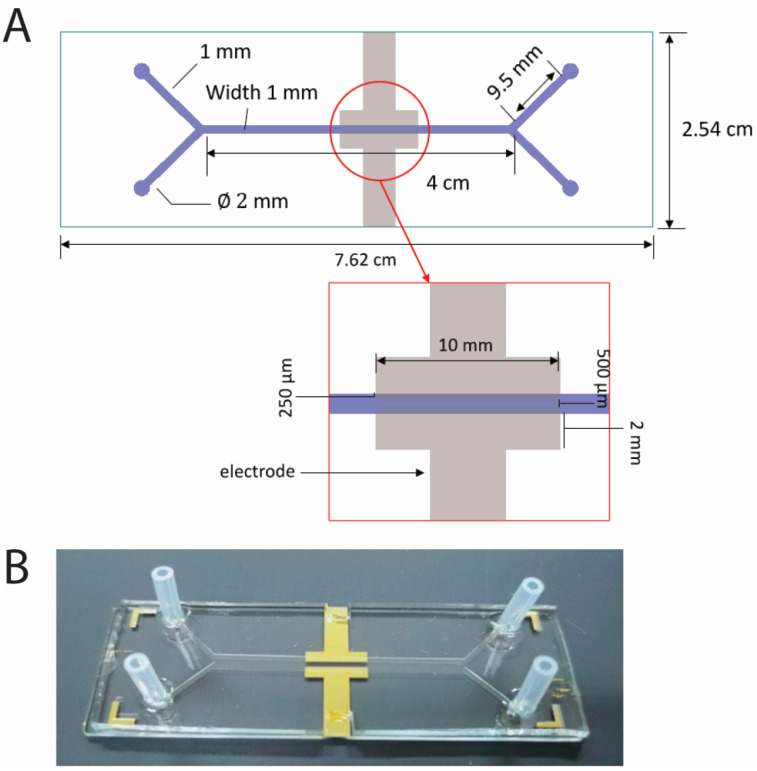
(**A**) Dimensions of microfluidic chip with two inlets and two outlets; (**B**) image of the microfluidic biofuel cell.

**Figure 5:**
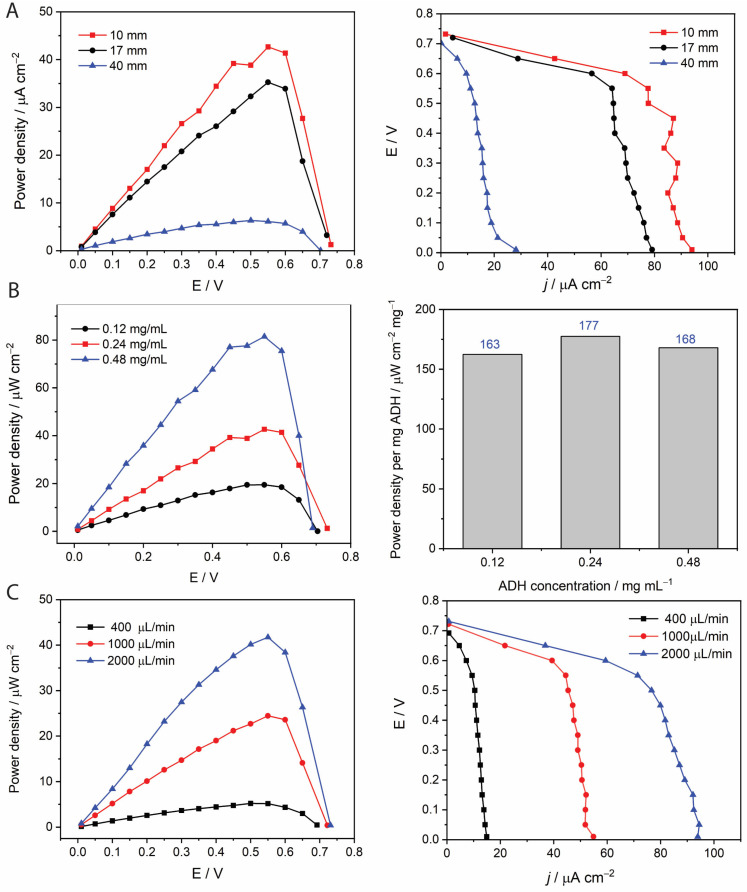
Effect of varying (**A**) the electrode length (10–40 mm), (**B**) the ADH concentration (0.12–0.48 mg/mL) and (**C**) the flow rate (400–2000 µL/min) on the membraneless-MBFC performance. Unless specified otherwise, the anolyte contained 0.24 mg/mL ADH, 1.1 mM TBO, 2.5 mM NAD^+^, and 0.57 mM EtOH; the catholyte contained 0.5 mg/mL HRP, 5.0 mM ABTS, and 10 mM H_2_O_2_, and the two streams were supplied at a flow rate of 1000 µL/min each.

We investigated the performance of different microfluidic cells (Figure 4) for varying electrode lengths (10–40 mm), while maintaining the microchannel width constant across designs (1 mm). The power density curves obtained are shown in Figure 5A. A significant decline in power density and current density is observed with increasing the electrode length. On the other hand, the total power and density generated remained almost constant (Figure S2). This trend can be expected by depletion of substrate along the electrode length, especially in the vicinity of the electrode substrate [33].

The effect of alcohol dehydrogenase (ADH) concentration in the anolyte was investigated by varying its concentration from 0.12 to 0.48 mg/mL. An increase in ADH concentration resulted in higher power density, attributed to the greater availability of the enzyme for catalyzing ethanol oxidation (Figure 5B). Although the highest power density (approximately 80 µW/cm^2^) was achieved at 0.48 mg/mL ADH, this concentration also required a substantial amount of enzyme. Consequently, an ADH concentration of 0.24 mg/mL was selected for further experiments, providing a sufficient power output and reasonable enzyme consumption.

The effect of varying the flow rate (400, 1000. and 2000 µL/min) on the power density supplied by the MBFC is shown in Figure 5C. The power density improved significantly as the flow rate increased. The OCV output varied from 0.70 to 0.74 V. The high power output at the high flow rate may be attributed to improved mass transport to and from the electrode surface with higher Reynolds numbers [13].

### 2.3. Impact of the Electrolyte Viscosity on the MBFC Performance

**Figure 6:**
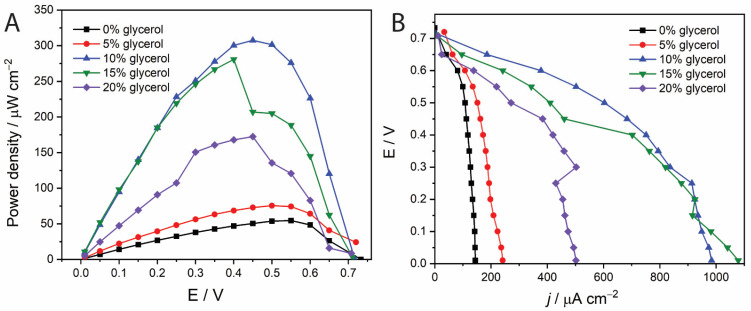
Impact of glycerol concentration (% *v*/*v*) on (**A**) cell power output and (**B**) cell polarization curve. The anolyte contained 0.24 mg/mL ADH, 1.1 mM TBO, 2.5 mM NAD^+^, and 0.57 mM EtOH, and the catholyte contained 0.5 mg/mL HRP, 5.0 mM ABTS, and 10 mM H_2_O_2_. The tests were conducted at a total flow rate of anolyte and catholyte of 2000 µL/min. Both anolyte and catholyte streams had the same glycerol concentration.

Next, we studied the impact of viscosity on the power output of the MBFC by adding glycerol to the electrolytes. We verified in blank experiments that glycerol, unlike ethanol, is not oxidized by yeast ADH. Power density and polarization profiles for electrolytes with varying glycerol concentrations are presented in Figure 6.

When the glycerol concentration in both anolyte and catholyte was increased from 0 to 10% *v*/*v*, a significant rise in power density was observed, correlating with an increase in viscosity. The maximum power output reached 307 µW cm^−2^ with 10 % *v*/*v* glycerol, which was ca. 6 times higher than that in the absence of glycerol. For even higher glycerol concentrations, however, the power density started to decrease.

We interpret that increasing the viscosity may reduce cross-over mixing between the electrolytes, improving performance. However, reducing the Reynolds number (Table 1) can also slow down mass transfer to and from the electrode surface, which can negatively impact performance (see Section 2.2). Thus, an optimal glycerol concentration exists. Our results demonstrate that a small addition of an inexpensive viscosity modifier like glycerol can lead to a substantial boost in the membraneless-MBFC performance.

### 2.4. Calibration of the MBFC-Based Ethanol Biosensor

**Figure 7:**
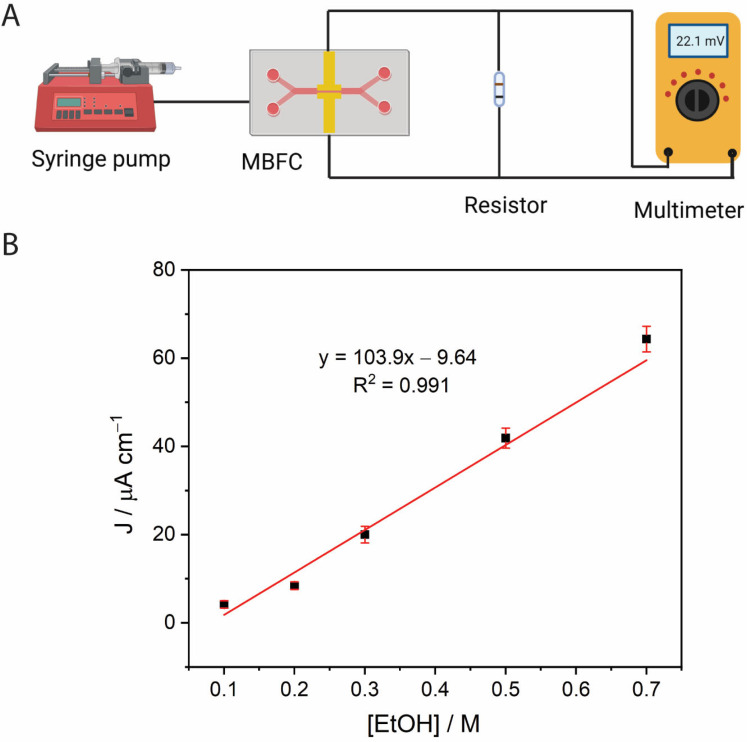
(**A**) Ethanol biosensor based on a microfluidic BFC setup for the determination of ethanol in samples. The microfluidic BFC is connected parallel to a resistor using a digital multimeter as readout. (**B**) Current density response to ethanol concentrations ranging from 0.1 to 0.7 M Measurement of the microfluidic BFC and the 24 kΩ resistor, a total flow rate of 2000 µL/min and using a multimeter as readout (n = 3).

A calibration curve for ethanol determination was constructed using a membraneless-MBFC with a digital multimeter as a readout. As shown in Figure 7A, the MBFC was connected to a resistor as an electrical load and no voltage was applied, eliminating the need for a potentiostat for signal readout. The cell voltage values obtained for each ethanol concentration were converted to current values (*i*) using Ohm’s law (*V = IR*) and then to current density (*j*) using the nominal electrode surface area.

The effect of the electrical resistance (10, 24, 50, and 100 kΩ) was investigated. The highest sensor sensitivity was observed with the 24 kΩ resistor (Figure S3), which was selected for further study. Under the selected conditions, the current density obtained from the BFC showed a linear dependence on ethanol concentrations ranging from 0.10 to 0.70 M (Figure 7B). Furthermore, we investigated the reproducibility of five different membraneless-MBFCs using 0.5 M ethanol. The current densities obtained (39.80 µA cm^−2^) demonstrated good reproducibility with a relative standard deviation (%RSD) of 3.45%.

The time needed for one measurement with the proposed system was 30 s. Furthermore, the proposed system exhibited a high specificity for ethanol. The addition of 0.3 M methanol to the 0.5 M ethanol anolyte solution resulted in a −2.46% change in signal, indicating negligible interference from methanol. Table 2 compares selected ethanol BFCs and MBFCs for power generation and sensing applications.

### 2.5. Application of the MBFC Ethanol Biosensor

A commercial liquor was diluted 20-fold in anolyte (200 µL liquor sample into 3.80 mL anolyte). The concentration of ethanol in the liquor samples was determined by using the external calibration curve. The concentrations determined using the MBFC biosensor were compared to independent determinations by gas chromatography (GC), obtaining excellent agreement between both methods (Table 3).

## 3. Materials and Methods

### 3.1. Chemicals and Materials

2,2′-Azino-bis-(3-ethylbenzothiazoline-6-sulfonic acid) ammonium salt (ABTS), toluidine blue (TBO), β-nicotinamide adenine dinucleotide in oxidized form (NAD^+^), alcohol dehydrogenase (ADH) from yeast (210 Units/mg), horseradish peroxidase (HRP, 174 Units/mg), and hydrogen peroxide (H_2_O_2_, 35%) were purchased from TCI Chemicals (Saitama, Japan). Ethanol (EtOH, 99.9%) was obtained from QRëC (New Zealand). A quantity of 0.1 M phosphate buffer pH 7.4 was prepared from di-sodium hydrogen phosphate anhydrous (Na_2_HPO_4_) and sodium di-hydrogen phosphate dihydrate (NaH_2_PO_4_·2H_2_O) from Kemaus (New South Wales, Australia). All aqueous solutions used in this work were prepared using DI water. MIR 726 developer and AZ^®^ 1512 were purchased from Merck Performance Materials GmbH (Wiesbaden, Germany). Poly(dimethylsiloxane) (PDMS) was prepared from the SYLGARD™184 Silicone Elastomer Kit purchased from Dow Chemical Company (Wiesbaden, Germany).

### 3.2. Fabrication of Microfluidic Biofuel Cells with Microchannel

The procedure for fabricating the microfluidic biofuel cell was adapted from a previous work by Zebda et al. [47] The microfluidic chip was designed to have a Y-shape with two inlets and two outlets. The channel dimensions were as follows: length (L) = 40 mm, width (w) = 1 mm, and height (h) = 100 µm. The dimensions of the electrodes were length (L) = 10 mm and width (w) = 0.25 mm. The distance between the electrodes and between the electrodes and the channel walls was 500 µm, as shown in Figure 4A.

Microfluidic chips were fabricated by photolithography (UV-Lithography) at the Synchrotron Light Research Institute, Beamline 6 Deep X-ray lithography. The process began with the design of a photomask (UV mask) using Clewin ver. 4.0 software. Once designed, the photomask was printed onto a transparent sheet, as shown in Figure S4A. Next, a 2 µm thick layer of AZ^®^ 1512 photoresist was coated onto a clean glass slide using a spin coater and then baked dry at 95 °C for 5 min. The photomask was then placed on the photoresist and exposed to 128 mJ/cm^2^ of UV radiation (365–405 nm), as shown in Figure S4B. After exposure, the photoresist was developed with MIR 726 developer. The exposed area of the photoresist was hardened, while the unexposed areas were washed away with MIR 726 developer, resulting in a patterned structure according to the designed photomask (Figure S4C). A 150 nm thick gold electrode layer was then deposited onto the glass slide via DC sputtering at a power of 75 W for 10 min (Figure S4D). Finally, the AZ^®^ 1512 photoresist was removed (Figure S4E) using acetone followed by isopropyl alcohol (IPA) and DI water.

The microchannel was designed using Clewin ver. 4.0 software to create a photomask (UV mask) as shown in Figure S5A. A 100 µm thick layer of SUEX™ photoresist (DJ MicroLaminates, Sudbury, MA, USA) was then coated onto a glass slide using a spin coater. The coated slide was baked at 65 °C for 5 min, then at 95 °C for 10 min, and allowed to cool to room temperature. The photomask was placed on the photoresist and exposed to 656 mJ/cm^2^ of UV radiation, as shown in Figure S5B. The SUEX™ photoresist was then cross-linked by baking at 65 °C for 5 min and 95 °C for 10 min, followed by cooling to room temperature. Next, the pattern was developed using 2-(1-methoxy) propyl acetate 99% developer to obtain the SUEX™ microchannel mold, as shown in Figure S5C. The master mold was used to create a replica in PDMS (Figure S5D) by curing at 70 °C for 1 h. After cooling, the PDMS replica was carefully peeled away, revealing the microchannel (Figure S5E). The PDMS replica was then treated with oxygen plasma (200 W for 1 min) to facilitate bonding to the glass slide containing the electrodes. Inlet ports were created using a 1.2 mm diameter punch to accommodate Teflon tubing (Figure S5F).

### 3.3. Electrochemical Measurements of Anode, Cathode, and 2-Compartment BFC

The formal potentials of the anode and cathode were estimated from the open-circuit potentials (OCPs) of the anolyte and catholyte measured in a separate three-electrode setup. The setup consisted of a 2 mm gold disk electrode as a working electrode, a Pt sheet electrode (1 cm^2^) as a counter electrode, and an Ag/AgCl 3 M KCl as a reference electrode. The anolyte and catholyte had a volume of 10 mL each and were stirred at 400 rpm during measurements. The OCPs of the anode and cathode were evaluated with and without the substrate in air-equilibrated electrolyte.

The performance of the ethanol/hydrogen peroxide BFC was evaluated using a two-electrode electrochemical setup. Gold disk electrodes were employed as the anode and cathode, and placed in separate compartments of a cell divided by a Nafion 117 membrane. Each compartment was filled with 10 mL of the respective electrolyte. The anode was connected to the working electrode terminal of a potentiostat, while the cathode was connected to the combined reference/counter electrode terminal. Chronoamperometry was used to record the current profiles at operating voltages ranging from 0.010 to 0.62 V versus the OCV.

### 3.4. Electrochemical Measurements of the Membraneless-MBFC

The solutions employed in the electrochemical measurements were as follows. The anolyte consisted of ADH 50.4 Units/mL (0.24 mg/mL), 2.5 mM NAD^+^, 1.1 mM TBO, and EtOH at various concentrations, whereas the catholyte consisted of HRP 87 Units/mL (0.5 mg/mL), 5.0 mM ABTS, and 10 mM H_2_O_2_. Both anolyte and catholyte were freshly prepared in phosphate buffer pH 7.4 and were air-equilibrated during the experiments.

To perform the microfluidic BFC experiments, a two-electrode electrochemical cell setup was required. A gold electrode for the anode was connected to a working electrode terminal of the potentiostat while another gold electrode was wired to the combined electrodes including a counter and reference electrode terminal. The solutions were delivered to the microfluidic cell by two separate programmable syringe pumps (NE-1000, New Era Pump Systems Inc., Farmingdale, NY, USA) at varying flow rates. A setup of the microfluidic BFC setup is illustrated in Figure 1A.

Chronoamperometric measurements were performed using an EmStat3^+^ blue potentiostat equipped with PSTrace 5.10 software (Palmsens, Houten, The Netherlands). Power curves were obtained from a sequence of potential chronoamperometry measurements at potentials from 0.010 to 0.73 V relative to the open-circuit voltage (OCV) of the BFC. The microfluidic BFC for ethanol sensing was demonstrated by connecting the anode and the cathode to a multimeter and an external resistor (24 kΩ) in parallel. The voltage outputs obtained at different ethanol concentrations in the anolyte were used to construct an external calibration curve. Liquor samples purchased from a local supermarket in Thailand were diluted 20-fold in the anolyte containing ADH and TBO in PBS buffer pH 7.4 to adjust the ethanol concentration to the calibration range prior to ethanol concentration analysis.

### 3.5. Quantification of Ethanol in Liquor Samples Using Gas Chromatography

Ethanol analysis was performed using a Shimadzu GC 2014 gas chromatograph (Canby, Oregon, USA) equipped with a flame ionization detector (FID) and an Rtx-Wax polyethylene glycol capillary column. Helium was used as the carrier gas at a flow rate of 1.0 mL min^−1^. A 1.00 µL injection volume was employed for all analyses. Samples and ethanol standards were prepared to contain 5% (*v*/*v*) n-butanol as an internal standard. Ethanol standards were prepared by diluting 99.9% (*v*/*v*) ethanol down to 0.2–1.0% (*v*/*v*) with deionized water. Samples were diluted 50 or 100 times with deionized water prior to analysis. The ethanol content in the samples was calculated from a linear calibration between the ratio of ethanol to n-butanol peak areas and the concentration of the ethanol in the standards.

## 4. Conclusions

This study demonstrates the development of a membraneless-MBFC for ethanol quantification, utilizing NAD-ADH and HRP enzymes in separate anolyte and catholyte solutions and redox mediators for efficient mediated electron transfer. The microfluidics chip enabled a laminar flow and eliminated the need for physical separation between the electrolytes. The study of some parameters including the electrode length and flow rate highlighted the key role of mass transport, which can be optimized to yield high power from the MBFC. Notably, we achieved a 6-fold improvement in power density output (307 µW cm^−2^) and an open-circuit voltage of 0.733 V by introducing 10% *v*/*v* glycerol as a viscosity modifier to the electrolyte. The increased viscosity may serve to reduce the cross-over mixing between anolyte and catholyte streams. The microfluidic biofuel cell was successfully applied as a MBFC-based sensor for the determination of ethanol in a liquor sample. This approach offers a promising solution for ethanol sensing and paves the way for further advancements in biofuel cell technology.

## Figures and Tables

**Table 1:** Reynolds number of the membraneless-MBFC with different glycerol concentration.

%Glycerol (*v*/*v*)	Dynamic Viscosity [44] (N·s/m^2^)	Reynolds Number
0.0	0.0008927	67.6
5.0	0.0010387	58.1
10.0	0.0012189	49.5
15.0	0.0014441	41.8
20.0	0.0017288	34.9

**Table 2:** Selected ethanol BFCs and MBFCs for power generation/sensing application.

Electrode Material	Application	Operation	Anode		Cathode		OCV (V)	Power Density (µW cm^−2^)	Ref.
			Enzyme	Mediator	Enzyme/Catalyst	Mediator			
anode: carbon ink microelectrode	Power generation	Flow/microchip (MBFC)	NAD-ADH	polyMG	Pt	−	0.34	53	[26]
Three-dimensional coralloid nitrogen doped hierarchical-micro/mesoporous carbons aerogels (3D-NHCAs)	Sensing	Wearable/flexible	AOx	−	BOx	−	0.62	1.9	[27]
anode: CNPs/PEI/Au cathode: CNPs/Au	Power generation	Flow/microfluidic (MBFC)	NAD-ADH	VK3	Laccase	ABTS	0.80	90	[33]
anode: GE cathode: MWCNTs-carbon SPE	Sensing	Immersion	NAD-ADH	PVP-TBO	AOx and HRP	−	0.66	8.9	[31]
SWCNTs/GCE	Power generation	Immersion	NAD-ADH	MG	BOx	−	0.53	11	[45]
anode: PAMAM/MWCNT/CC cathode: CC	Power generation	Immersion	NAD-ADH	polyMG	BOx	Poly-Os complex	0.226	21	[29]
anode: CP cathode: CGDE	Power generation	Immersion	NAD-ADH and NAD-AldDH	polyMG	Pt on Vulcan	−	0.54	186	[30]
anode: pyrene-TEMPO-COOH-MWCNTs/CC cathode: CGDE	Power generation	Immersion	OxDc/TEMPO	-	Pt on Vulcan	−	0.598	388 × 10^5^	[32]
anode: chitosan/CNTs-IL cathode: chitosan/ITO	Power generation	Immersion	NAD-ADH	Meldola blue	GOx/PB	−	0.53	37.5	[46]
Au electrode	Sensing	Flow/microfluidic (MBFC)	NAD-ADH	TBO	HRP	ABTS	0.74	75	This work

CC: carbon cloth, CP: carbon paper, CGDE: carbon gas diffusion electrode, GE: graphite electrode, SPE: Screen-printed electrode, GCE: glassy carbon electrode, ITO: indium tin oxide, MWCNTs: multi-walled carbon nanotubes, SWCNTs: single-walled carbon nanotubes, CNTs-IL: carbon nanotubes-ionic liquid, CNPs: carbon nanoparticles, PVP-TBO: Poly(4-vinylpyridine)-toluidine blue, PAMAM: polyamidoamine generation 4 dendrimer, MG: methylene green, polyMG: poly(methylene green), PB: Prussian blue, VK3: 2-methyl-1,4-naphthoquinone, PEI: poly ethyleneimine, ABTS = 2,2′-Azino-bis-(3-ethylbenzothiazoline-6-sulfonic acid ammonium, AOx: alcohol oxidase, BOx: birilubin oxidase, HRP: horseradish peroxidase, AldDH: aldehyde dehydrogenase, GOx: glucose oxidase, OxDc: Oxalate decarboxylase.

**Table 3:** Determination of ethanol in commercial liquor samples.

Sample	% Label (% *v*/*v*)	The Proposed Method (% *v*/*v*)	GC (% *v*/*v*)
Tawan dang, spirit	40	39.5 ± 1.2	39.0
Bangyikhan, spirit	40	40.1 ± 1.3	39.0
Niyom Thai, whisky	40	39.6 ± 1.6	40.6
Ruang khao, spirit	40	39.9 ± 1.3	38.9
Soju	16.5	15.6 ± 0.2	16.1

## Data Availability

The data that support the findings of this study are available from the corresponding authors upon reasonable request.

## Supplementary Materials

The following supporting information can be downloaded at: https://www.mdpi.com/article/10.3390/molecules30030673/s1. Figure S1: Effect of pH on ADH anode response. Figure S2: Effect of varying the electrode length (10–40 mm) on (A) current and (B) power output profiles of the membraneless-MBFC. Figure S3: Effect of resistance (10, 24, 50, and 100 kΩ) on the current density obtained from the membraneless-MBFC using electrolytes with 0.1–1.0 M ethanol and no glycerol. Figure S4: Fabrication process of the electrodes. Figure S5: Fabrication process of the membraneless-MBFC.
