# Supplementary material for: Small but Mighty: A Microfluidic Biofuel Cell-Based Biosensor for the Determination of Ethanol
Source: Molecules. 2025 Feb 3;30(3):673. doi: 10.3390/molecules30030673 (PMC11821114; doi:10.3390/molecules30030673)
Supplement: Supplementary file 1 [file molecules-30-00673-s001.zip › molecules-3347089-supplementary.pdf]

SUPPORTING INFORMATION

Small but Mighty: A Microfluidic Self-Powered Biosensor for the Determination of Ethanol

Jirawan Monkratok [1,2], Pattanaphong Janphuang [2], Kantapat Chansaenpak [3], Sireerat Lisnund [4], Vincent Blay [5,*] and Piyanut Pinyou [1,*]

[1] School of Chemistry, Institute of Science, Suranaree University of Technology, 111 University Ave., Nakhon Ratchasima 30000, Thailand; jirawan@g.sut.ac.th

[2] Synchrotron Light Research Institute (Public Organization), 111 University Ave., Nakhon Ratchasima 30000, Thailand; pattanaphong@slri.or.th

[3] National Nanotechnology Center, National Science and Technology Development Agency, Thailand Science Park, Pathum Thani 12120, Thailand; kantapat.cha@nanotec.or.th

[4] Department of Applied Chemistry, Faculty of Science and Liberal Arts, Rajamangala University of Technology Isan, 744, Suranarai Rd., Nakhon Ratchasima 30000, Thailand; sireerat.in@rmuti.ac.th

[5] Department of Microbiology and Environmental Toxicology, University of California at Santa Cruz, Santa Cruz, CA 95064, USA

* Correspondence: vroger@ucsc.edu (V.B.); piyanutp@sut.ac.th (P.P.)

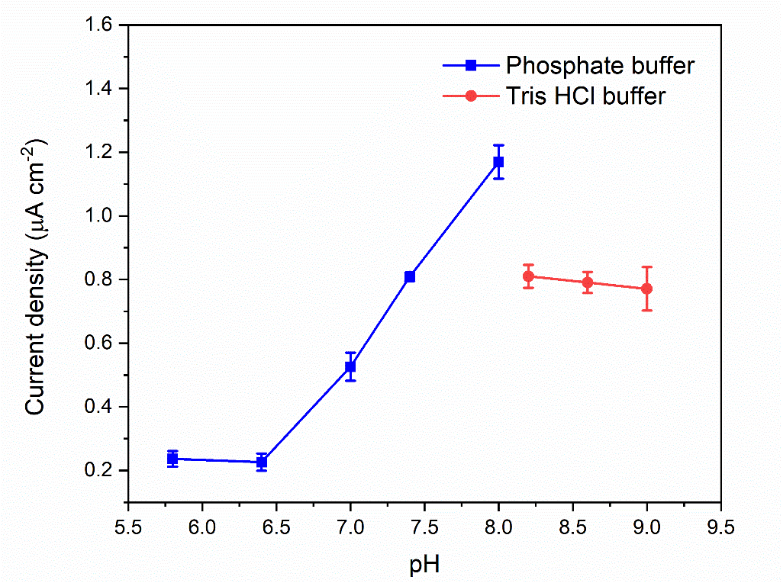

Figure S1. Effect of pH on ADH anode response. Current density values were obtained from 1.0 M ethanol in 0.1 M phosphate buffer pH 5.8-8 or 0.1 M Tris HCl buffer pH 8.2-9.0 containing 0.24 mg/mL ADH, 1.1 mM TBO and 2.5 mM NAD^+. Amperometry was conducted at a constant applied potential of 0.20 V vs. Ag using a 4 mm gold screen printed electrode as the working electrode (n= 3).

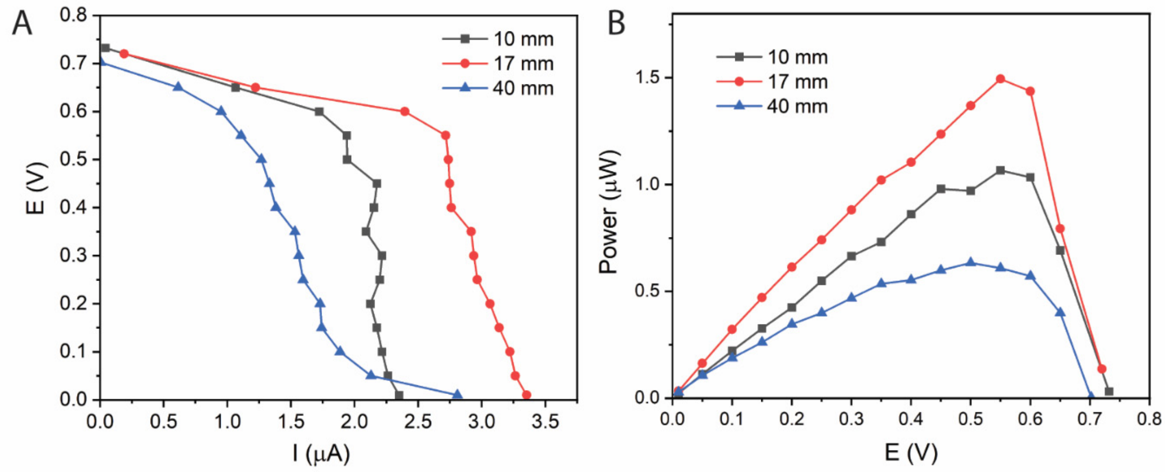

Figure S2. Effect of varying the electrode length (10-40 mm) on (A) current and (B) power output profiles of the membraneless-MBFC. The anolyte contained 0.24 mg/mL ADH, 1.1 mM TBO, 2.5 mM NAD^+**,** and 0.57 mM EtOH, the catholyte contained 0.5 mg/mL HRP, 5.0 mM ABTS and 10 mM H_2O_2, and each electrolyte solution was supplied at a flow rate of 1000 μL/min.

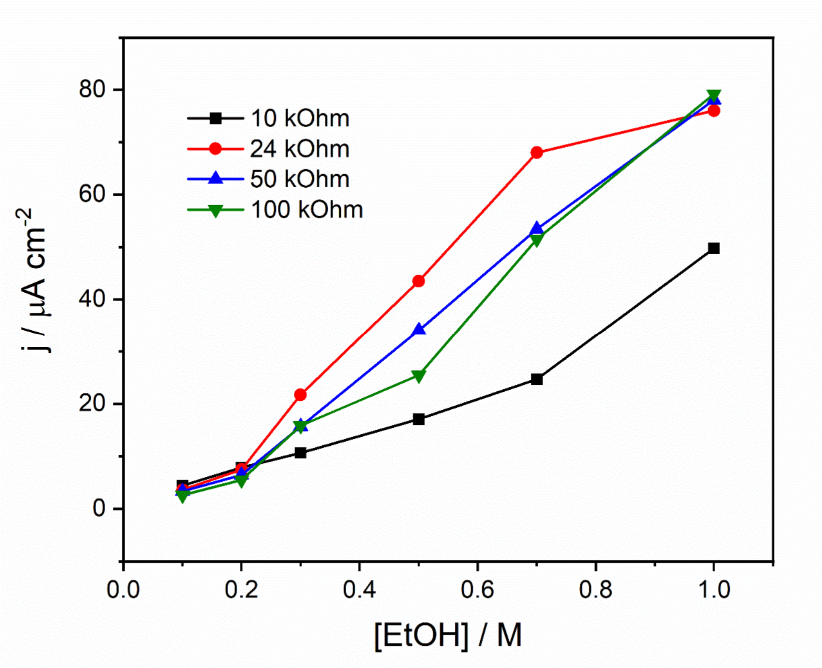

Figure S3. Effect of resistance (10, 24, 50, and 100 kΩ) on the current density obtained from the self-powered membraneless-MBFC using electrolytes with 0.1-1.0 M ethanol and no glycerol.

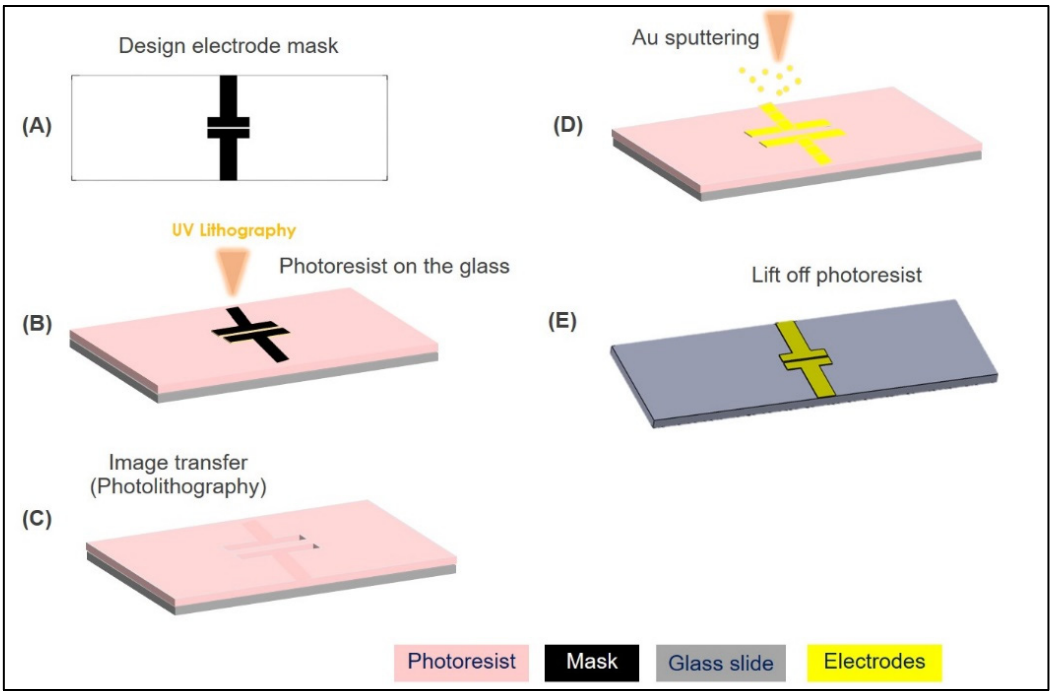

Figure S4. The fabrication process of electrode.

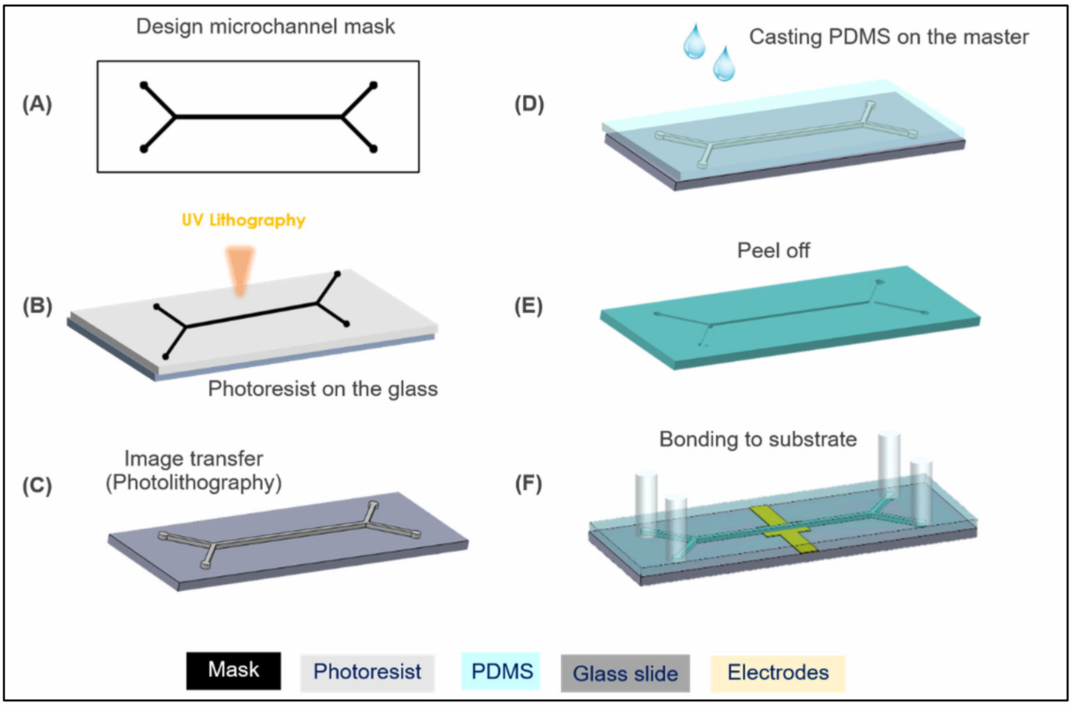

Figure S5. The fabrication process of the membraneless-MBFC.
